# MicroRNA signatures of endogenous Huntingtin CAG repeat expansion in mice

**DOI:** 10.1371/journal.pone.0190550

**Published:** 2018-01-11

**Authors:** Peter Langfelder, Fuying Gao, Nan Wang, David Howland, Seung Kwak, Thomas F. Vogt, Jeffrey S. Aaronson, Jim Rosinski, Giovanni Coppola, Steve Horvath, X. William Yang

**Affiliations:** 1 Department of Human Genetics, David Geffen School of Medicine at UCLA, Los Angeles, CA, United States of America; 2 Center for Neurobehavioral Genetics, Semel Institute for Neuroscience & Human Behavior, University of California Los Angeles (UCLA), Los Angeles, CA, United States of America; 3 Department of Psychiatry and Biobehavioral Sciences, David Geffen School of Medicine at UCLA, Los Angeles, CA, United States of America; 4 UCLA Brain Research Institute, Los Angeles, CA, United States of America; 5 CHDI Foundation/CHDI Management Inc., Princeton, NJ, United States of America; 6 Department of Neurology, David Geffen School of Medicine at UCLA, Los Angeles, CA, United States of America; 7 Department of Biostatistics, David Geffen School of Medicine at UCLA, Los Angeles, CA, United States of America; Emory University, UNITED STATES

## Abstract

In Huntington's disease (HD) patients and in model organisms, messenger RNA transcriptome has been extensively studied; in contrast, comparatively little is known about expression and potential role of microRNAs. Using RNA-sequencing, we have quantified microRNA expression in four brain regions and liver, at three different ages, from an allelic series of HD model mice with increasing CAG length in the endogenous Huntingtin gene. Our analyses reveal CAG length-dependent microRNA expression changes in brain, with 159 microRNAs selectively altered in striatum, 102 in cerebellum, 51 in hippocampus, and 45 in cortex. In contrast, a progressive CAG length-dependent microRNA dysregulation was not observed in liver. We further identify microRNAs whose transcriptomic response to CAG length expansion differs significantly among the brain regions and validate our findings in data from a second, independent cohort of mice. Using existing mRNA expression data from the same animals, we assess the possible relationships between microRNA and mRNA expression and highlight candidate microRNAs that are negatively correlated with, and whose predicted targets are enriched in, CAG-length dependent mRNA modules. Several of our top microRNAs (*Mir212*/*Mir132*, *Mir218*, *Mir128* and others) have been previously associated with aspects of neuronal development and survival. This study provides an extensive resource for CAG length-dependent changes in microRNA expression in disease-vulnerable and -resistant brain regions in HD mice, and provides new insights for further investigation of microRNAs in HD pathogenesis and therapeutics.

## Introduction

Huntington’s disease (HD) is a dominantly inherited neurodegenerative disorder clinically characterized by progressive movement disorder, cognitive dysfunction, and psychiatric impairment [1]. The hallmark of HD neuropathology is selective degeneration of striatal medium spiny neurons (MSNs), and, to a lesser extent, deep-layer cortical pyramidal neurons [2]. Currently there are no therapies to prevent the onset or slow the progression of HD.

HD is caused by a CAG trinucleotide repeat expansion encoding an elongated polyglutamine (polyQ) stretch near the N-terminus of Huntingtin (HTT) [3]. Unaffected individuals have CAG repeat lengths ranging from 6–35, and HD individuals have repeat lengths greater than 36 on one *HTT* allele, with the length of the CAG repeat inversely correlated with the age of disease onset [4,5]. Patients with CAG lengths in the 40s often have motor symptom onset in the fourth decade while repeat lengths greater than 60 lead to juvenile onset [6]. Patients with repeat lengths of 37–40 may have very late onset or no observed clinical symptoms. In contrast to age of clinical onset, the influence of CAG repeat length on disease progression is more modest [6], suggesting an important impact of CAG length in early disease pathogenesis [7]. Recent imaging studies of HD patients suggest that CAG repeat length correlates with caudate atrophy [8] and that combined CAG repeat length and age is a useful predictor of many clinical outcomes in HD [6]. Overall, HD patient studies underscore a critical role of CAG repeat length in the onset of HD pathogenesis. The graded impact of CAG length on HD symptomatic onset led to the “polyQ molecular trigger” hypothesis, which suggests polyQ expansion in HTT leads to subtle repeat length-dependent graded molecular changes in vulnerable neuronal cells that act in a profound and dominant fashion to initiate the disease [7].

Multiple studies have examined mRNA expression signatures of HD in post-mortem human brains as well as in multiple mouse models. In contrast, comparatively little is known about changes in microRNA expression in HD patients or in animal models. Multiple microRNAs have been implicated in a handful of small studies performed on postmortem human tissue [9] and model organisms [10,11]. A larger recent study examined microRNA expression in HD patients, pre-symptomatic carriers and controls in human cortex and found multiple differentially expressed microRNAs, some of which modulate neuron survival [12].

Here we studied microRNAs dysregulated in an *Htt* CAG length- and age-dependent fashion across striatum, cortex, cerebellum, hippocampus, and liver of HD mouse models [13,14]. We generated and analyzed microRNA expression data from a series of murine huntingtin (*Htt*) knock-in (KI) models across 3 time points to identify individual microRNAs whose expression changes progressively with CAG length and age. The data we generated are publically available for additional mining and modeling through GEO and the HDinHD portal (www.HDinHD.org).

## Results

### Longitudinal microRNA analysis of brain and liver of HD allelic series mice

Using deep microRNA sequencing, we profiled the striatum, cortex, cerebellum, hippocampus and liver of 2-, 6-, and 10-month (denoted 2m, 6m, 10m) old mice that express one wild-type endogenous *Htt* allele and a second engineered *Htt* allele with a knock-in of a human *HTT* exon1 carrying one of 7 different CAG repeat lengths (denoted Q in the mouse strain labels). In the first phase of the study, we sequenced Q20, Q80, Q92, Q111, Q140, and Q175 plus wild type (WT) control littermates of the Q20 mice. These 6 Q-length *Htt* knockin lines include those that exhibit behavioral and physiological phenotypes within the mouse lifespan (Q140 and Q175), as well as those with modest (Q111) and no observable phenotypes but with molecular pathogenic signatures (Q92 and Q80) [15,16]. The Q20 mice are considered controls since the repeat length approximates the average human *HTT* repeat length (the mouse *Htt* gene has a CAG length of 7). In a second batch designed to add a Q50 allele, we sequenced Q20, Q50, Q92 and Q140 mice at age 6 and 10 months, plus WT littermates of the Q20 and Q50 lines. In each batch, we sequenced 8 animals per genotype and age for each tissue. A detailed analysis of the mRNA data from the first phase of this allelic series of *Htt* KI mice is described in [14]; here we focus on the analyses of the microRNA data.

**Fig 1:**
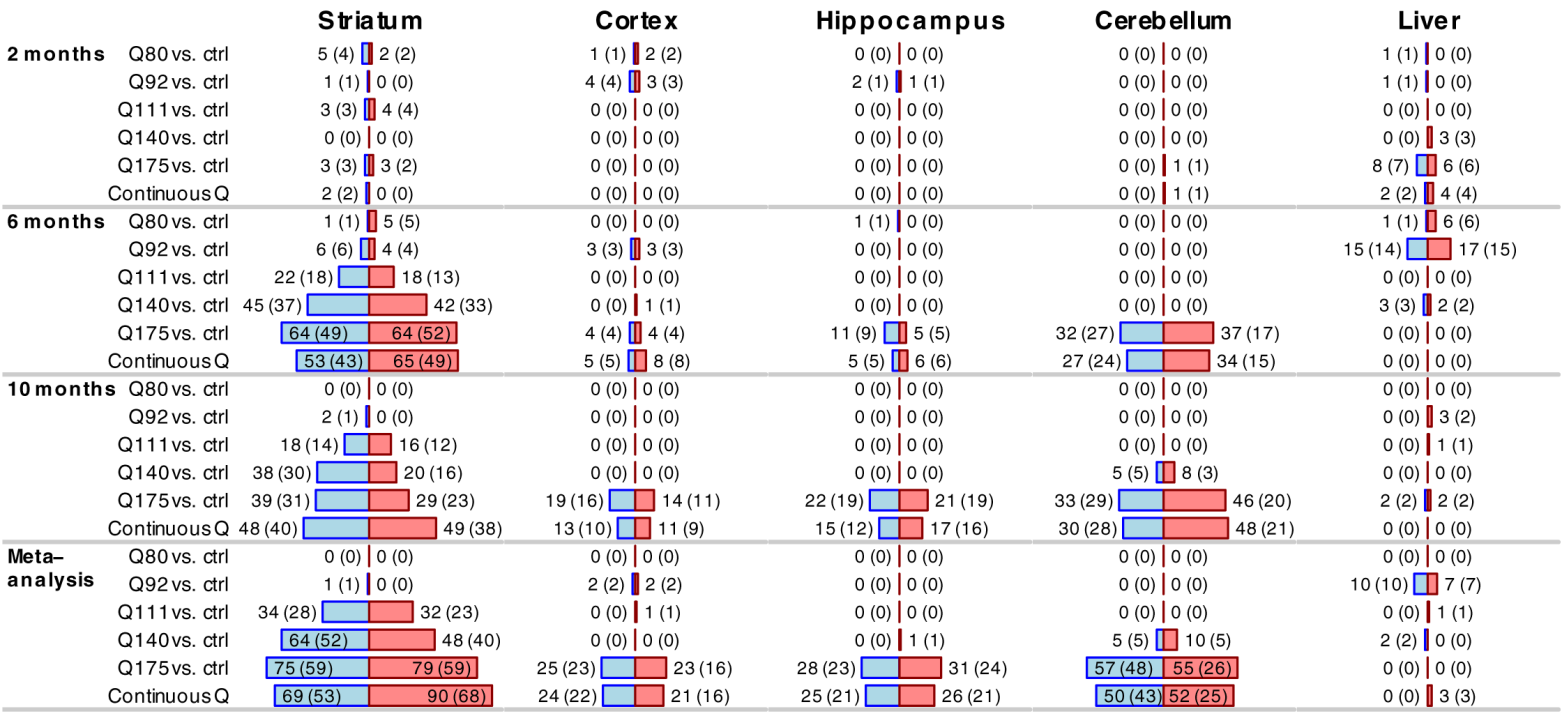
Differential expression analysis with respect to CAG length in Series 1. Bars show numbers of significantly (FDR<0.05) differentially expressed microRNAs in the 5 tissues studied in Series 1; the numbers are also shown next to each bar. The bars labeled Continuous Q represent numbers of microRNAs significantly associated with CAG length (Q) treated as a numeric variable. Numbers in parentheses represent the counts of distinct microRNA clusters in which the significant microRNAs fall into. Blue and red bars represent microRNAs significantly down- and up-regulated, respectively, with increasing CAG length (Q).

For each tissue and timepoint, we carried out differential expression (DE) analysis between mice with mutant CAG repeat lengths (Q50 and higher) and the Q20 and WT controls, as well as an analysis of association with CAG length as a numeric variable. We find robust age- and CAG length-dependent increases in differentially expressed microRNAs in the striatum and to a lesser degree in the cortex, cerebellum and hippocampus, while such CAG length-dependent differential expression is not a feature of the liver transcriptome (Fig 1; S1 Table).

To identify the microRNAs with the strongest evidence of DE across the 6- and 10-month points, we combined the DE statistics across the two time points in each tissue using meta-analysis. In the striatum, the meta-analysis identified 159 microRNAs associated with Q as a continuous variable at the FDR<0.05 significance level. Smaller numbers have been identified in cerebellum (102), hippocampus (51) and cortex (45). Only 3 microRNAs passed the meta-analysis FDR<0.05 threshold for association with Q in liver. These numbers are consistent with striatum being the brain region most affected by the *Htt* mutation in HD. The brain region with the second highest number of DE microRNAs is cerebellum, followed by hippocampus and cortex. Interestingly, while cerebellum is known to be relatively modestly affected in adult-onset HD, this brain region is known to be affected in Juvenile HD [17]. The microRNA transcriptional response to *Htt* knock-in mice, at least in the high CAG lengths, is very profound and consistent with the pathological findings in the patients.

At 2m of age we identified a modest number of DE microRNAs across most of the tissues, but the observed differential expression of these microRNAs does not appear to be progressive with increasing CAG length. In contrast, in the 6 and 10-month striatum we found a relatively large number of DE microRNAs that increases with CAG length. The trend is especially striking in 10-month striatum, where we found no DE microRNAs in Q80, 2 DE microRNAs in Q92, 34 DE microRNAs in Q111, 58 in Q140, 68 in Q175, and 97 microRNAs were significantly associated with Q viewed as a numeric variable. The sets of DE microRNAs in striatum overlap strongly between different comparisons as well as between 6 and 10 months (S1 Fig); for example, of the 40 and 34 DE microRNAs in Q111 samples at 6 and 10 months, 18 are common (hypergeometric test P = 9×10^−13^). The overlap of DE microRNAs observed in the 2-month Q175 and 6 and 10 month Q175 is also statistically significant: of the 6 significant microRNAs in 2 month Q175, 5 are also significantly DE in 6 and 10 month Q175 samples (P = 7×10^−3^ and 3×10^−4^ with 6 and 10 month DE microRNAs, respectively).

In cerebellum, significantly DE microRNAs were only detected in Q140 (13 microRNAs at 10 months) and Q175 lines (1, 69 and 79 microRNAs at 2, 6 and 10 months, respectively) as well as in association testing with Q as a numeric variable (1, 61 and 78 microRNAs at 2, 6 and 10 months). In cortex, most of the DE microRNAs were found in Q175 samples: 8 at 6m and 33 at 10m, with 5 microRNAs common (hypergeometric test p-value P = 6×10^−5^); only a small number of microRNAs were differentially expressed in lower CAG length samples (3 and 7 in Q80 and Q92 at 2 months, 6 in Q92 at 6 months). In hippocampus, we similarly found most DE microRNAs in Q175 samples: 16 and 43 at 6 and 10 months, respectively, 13 of which are common (hypergeometric test P = 2×10^−12^).

Liver samples show the most DE microRNAs at 6m and much fewer at 2m and 10m. In contrast to the striatum and cortex, the numbers of DE genes at 6m do not exhibit a trend of correlation with CAG length: The most robust differential expression is observed in Q92 mice (32 DE microRNAs), followed by Q80 (7 DE microRNAs), and Q140 (5 DE microRNAs). No significantly DE microRNAs were found in 6-month Q111 and Q175 samples. Finally, very few if any DE microRNAs were found in 10m liver across all genotypes.

Relatively little is known about possible brain-related functions of most of the top DE microRNAs identified in our analyses. We have carried out a GO enrichment analysis on sets of differentially expressed microRNAs but found no GO terms whose enrichment p-values are significant or at least suggestive after multiple testing correction. This could be due to the fact that only a relatively small fraction (40%) of the 480 microRNAs retained in our analysis are annotated in GO. Nevertheless, there are several individual microRNAs that have been implicated in potentially relevant pathways and processes. The top striatum hits include the downregulated *Mir212*/*Mir132* cluster whose knockout has been implicated in impaired synaptic function [18] and down-regulation in neuronal death via the PTEN/FOXO3a signaling pathway [19]. *Mir132* is required for normal dendrite maturation in newborn neurons in the adult hippocampus and may participate in other examples of CREB-mediated signaling [20]. Moreover, *Mir132* is induced by neuronal activities [21] and is implicated in regulating the function of the chromatin regulator MeCP2 [22]. The *Mir212*/*Mir132* cluster has also been implicated in regulation of aging-related processes via interaction with FOXO3 [23]. Several DE microRNAs have been implicated in the broad processes of neuron differentiation and maturation. *Mir218* is strongly downregulated in the striatum and has been implicated in establishing motor neuron fate as a downstream effector of *Isl1-Lhx3* [24]. Interestingly, expression of the *Isl1* gene increases significantly with CAG length in the same samples (Supplementary Table S1 in [14]) while *Lhx3* shows minimal expression. *Mir128* has been implicated in regulation of motor behavior by modulating neuronal signaling networks and excitability [25] and in regulating cortical lamination as well as for the development of neuronal morphology and intrinsic excitability [26].

### Validation of *Htt* CAG-length dependent microRNA response in different tissues using an independent RNA-sequencing series

To validate our results and explore effects of lower CAG lengths (i.e., Q50), we sequenced short RNA in 6- and 10-month striatum, cortex, cerebellum and liver samples from an independent set of Q20, Q50, Q92 and Q140 mice plus the WT littermates of the Q20 and Q50 lines. In this data set, referred to as Series 2, we again sequenced 8 samples (4 male and 4 female) per genotype, tissue and age. We analyzed these data using the same approach as the Series 1 data, testing differential expression between the samples with CAG length ≥50 and the controls (WT and Q20 samples), as well as testing the association between expression and Q considered as a numeric trait.

**Fig 2:**
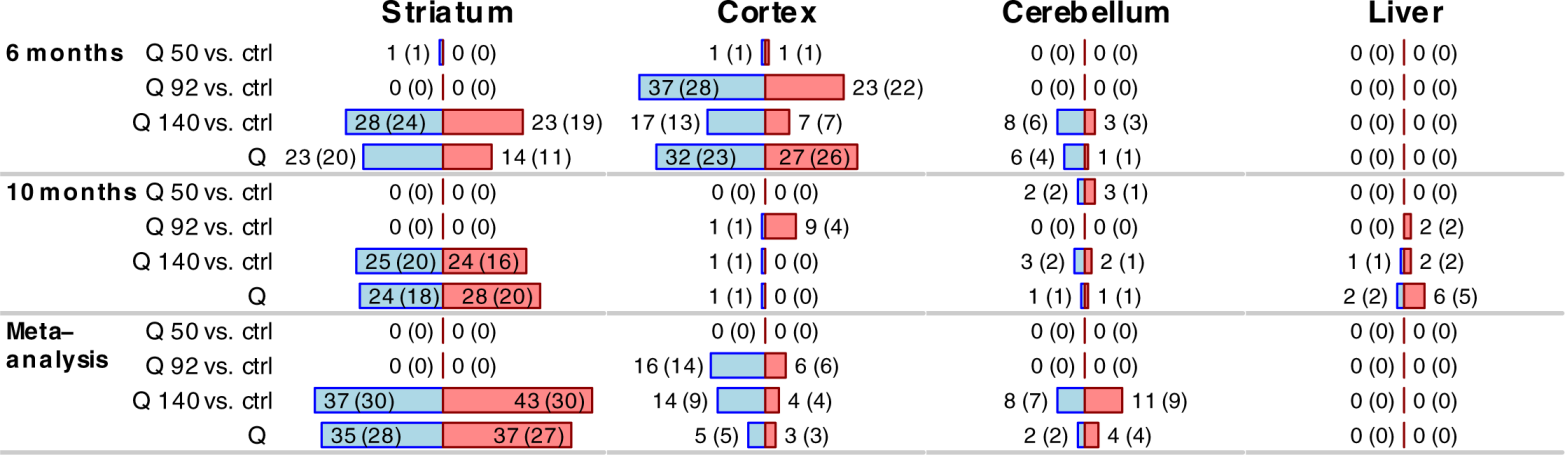
Differential expression analysis with respect to CAG length in Series 2. Bars show numbers of significantly (FDR<0.05) differentially expressed microRNAs in the 4 tissues studied in Series 2; the numbers are also shown next to each bar. Numbers in parentheses represent the counts of distinct microRNA clusters in which the significant microRNAs fall into. Blue and red bars represent microRNAs significantly down- and up-regulated, respectively, with increasing CAG length (Q).

We have found a small number of microRNAs with DE between Q50 and control samples that were significant in 6-month cortex (2 microRNAs) and in 10-month cerebellum (5 microRNAs) but none of the associations were significant in the meta-analysis of 6- and 10-month samples (Fig 2 and S1 Table). Similar observations apply also to the Q80 samples in Series 1, leading us to conclude that microRNA transcriptional changes in these two models (Q50 and Q80) are too weak to be reliably identified in our data.

To assess the concordance of DE between Series 1 and Series 2, we compared meta-analysis significance statistics for binary DE comparisons present in both series (Q92 and Q140) as well as continuous Q (S2 Fig). For Q92 vs. controls, we observe moderate concordance (correlation r = 0.29, permutation p-value p = 0.01, Methods) between Series 1 and Series 2 for striatum and statistically non-significant concordance in cortex, cerebellum and liver. In Q140 samples, we observe strong concordance between series 1 and 2 in striatum (r = 0.76, p = 3.7×10^−16^) and cerebellum (r = 0.53, p = 1.6×10^−7^), moderate concordance in cortex (r = 0.36, p = 0.004) and non-significant concordance in liver. For Q as a numeric variable, the concordance is strong in all 3 brain tissues (all correlation above 0.39, p<10^−5^) and moderate in liver (r = 0.28, p = 0.003).

**Fig 3:**
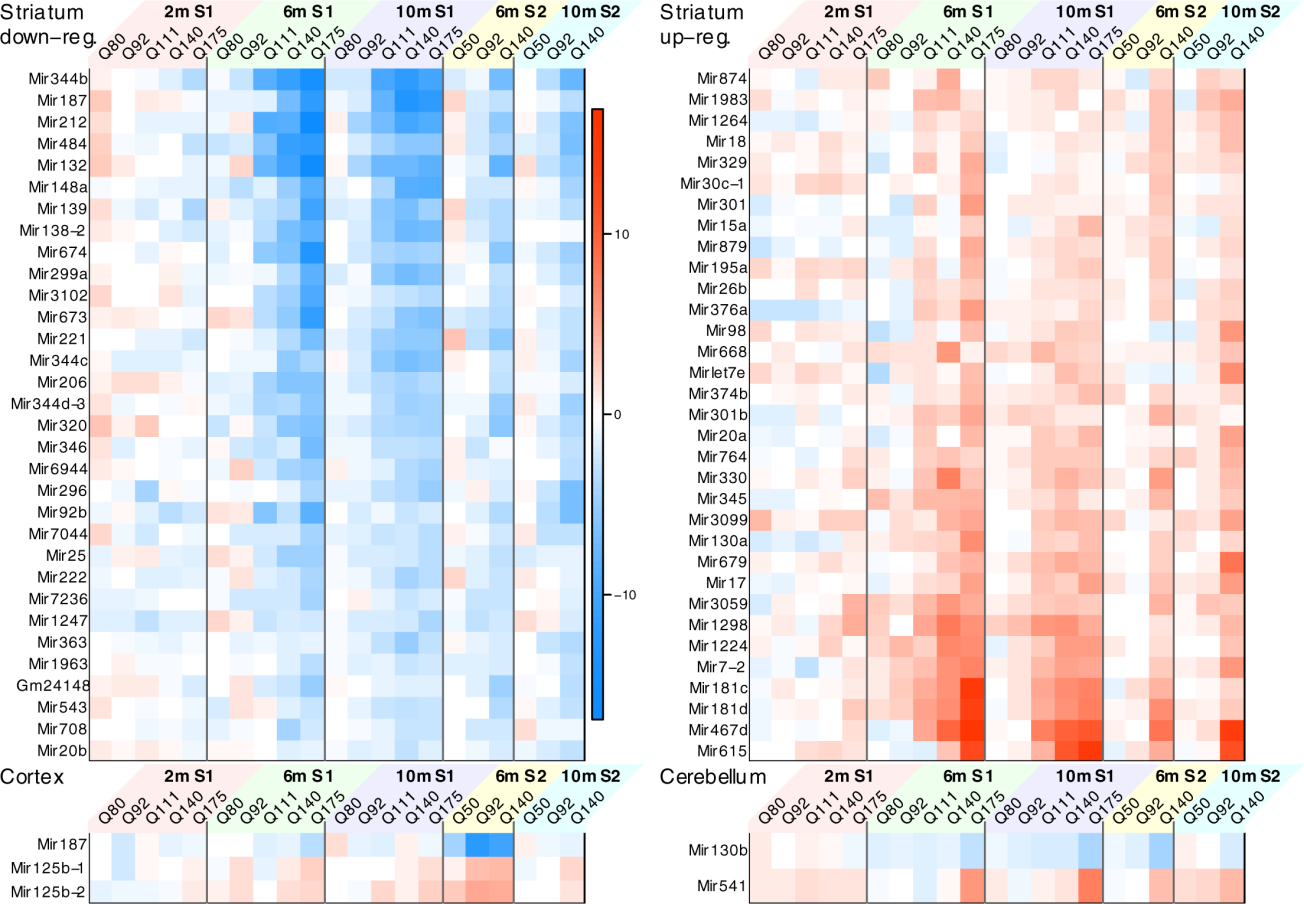
MicroRNA whose DE validates in Series 1 and Series 2. The heatmap represents differential expression Z statistics in all binary genotype comparisons for those microRNAs whose association with Q (as a numeric variable) passes the threshold FDR<0.05 in both Series 1 and Series 2. Top left and right panels show validated down- and up-regulated striatum microRNAs, respectively; bottom left and right panels show all validated microRNAs in cortex and cerebellum, respectively.

Another way to assess concordance is to evaluate the proportion of microRNAs called significantly associated with Q (at FDR<0.05 level) in Series 1 that are also significantly associated with Q in Series 2 (S2 Table). Of the 159 microRNAs significant for Q in Series 1 striatum, 93 (or 58%) and 65 (41%) are also associated with Q in Series 2 at the levels p<0.05 and FDR<0.05, respectively. In cortex, of the 45 microRNAs significant for Q in Series 1, 14 (32%) and 3 (7%) are also associated with Q in Series 2 at p<0.05 and FDR<0.05, respectively. In cerebellum, of the 102 microRNAs significant for Q in Series 1, 28 (27%) and 2 (2%) are also associated with Q in Series 2 at p<0.05 and FDR<0.05, respectively. Heatmaps of differential expression of validated microRNAs are shown in Fig 3.

### Cross-tissue comparisons of microRNA transcriptional response to CAG length expansion

To gain insight into global tissue similarities and differences of microRNA transcriptome response to polyQ expansion within HTT, we evaluated the concordance of differential expression among the various tissue and time point combinations. To that end, we represented each tissue by a vector of Z statistics from the analysis of association of microRNA profiles with Q as a numeric variable. Thus, *Htt* CAG length expansion has a similar effect on two tissues (or at two different time points) if their corresponding vectors of Z statistics are highly positively correlated. A heatmap of the correlations is shown in Fig 3 (the corresponding statistical significance is shown in S3 Fig) and provides several layers of information. The clustering of data sets by the similarity of the significance statistics groups together the striatum sets in a single relatively tight cluster and cerebellum data sets in another relatively tight cluster. First, the striatum in Series 1 and 2 at 6- and 10-months form a tight cluster, which is closely clustered with cortex and hippocampus data set (6 and 10 months) cluster from Series 1 and cortex 10-month from Series 2. This suggests that there is a broad overlap of cortical and striatal microRNA response to *Htt* CAG length expansion in allelic series mice. Interestingly, the striatum 2-month from series 1 already clustered with the broader 6- and 10-month striatum/cortex/hippocampus cluster, implying that the Q-length dependent microRNA dysregulation occurs earliest in the striatum. Second, the cerebellum from both series showed tight but distinct clusters form the striatum/cortex/hippocampus cluster, showing distinct cerebellar Q-length dependent microRNA expression. Finally, the 6- and 10-month liver data sets appear separate from all other data sets.

**Fig 4:**
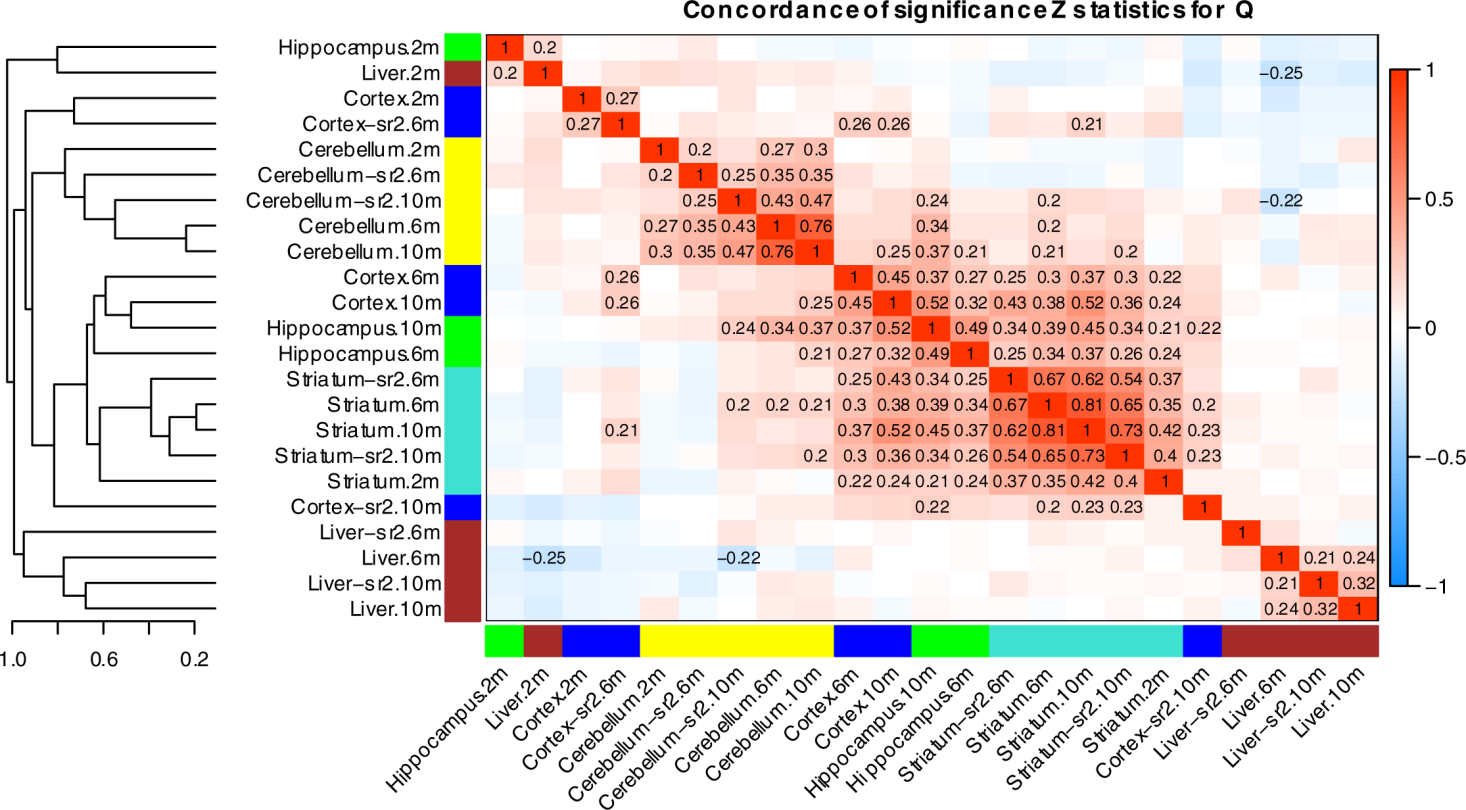
Concordance of differential expression across the 22 tissue/time point combinations. To generate this heatmap, we have arranged the miRNA significance Z statistics as a long vector in each of the 22 data sets (individual tissue, time point and series), and calculated correlations of these vectors. The correlations measure how similar the tissues are in their miRNA transcriptomic response to the CAG length mutation. Datasets are ordered by hierarchical clustering with the clustering tree shown on the left. Colors on the heatmap margins indicate tissue (turquoise, striatum; blue, cortex; green, hippocampus; yellow, cerebellum; brown, liver). Only correlations whose absolute value is above 0.20 are shown explicitly.

Consistent with the notion that microRNA responses are broadly distinct across all four brain regions, we only identified three microRNAs (*Mir484*, *Mir212*, and *Mir6944*) that are commonly dysregulated across all 4 brain regions. Using the 6- and 10-month meta-analysis statistics, we found a single microRNA, *Mir484*, that is differentially expressed (downregulated with increasing CAG length) across all 5 tissues in Series 1 and striatum in Series 2 at the p<0.05 threshold and changes in the same direction with Q across all 9 data sets (in Series 2 cortex and liver, the p-values are below 0.1 (suggestive) but above 0.2 in series 2 cerebellum). The non-significant results in cortex, cerebellum and liver in Series 2 may be due to the lack of Q175 samples in Series 2. Interestingly, *Mir484* has been implicated in suppressing translation of mitochondrial fission protein FIS1 and inhibited FIS1-mediated fission and apoptosis in cardiomyocytes and adrenocortical cancer cells [27]. Further studies are needed to determine whether the decreased expression of *Mir484* alters Fis1 levels in different brain regions in HD knockin mice and contributes to dysregulated mitochondrial dynamics in HD cells [28,29].

### MicroRNAs whose transcriptomic response to CAG length differs significantly between striatum, cortex and cerebellum

HD is characterized by selective neuronal vulnerability, with the striatal and deep layer cortical neurons most susceptible to degeneration; in contrast, neurons in other brain regions (e.g. cerebellum) and non-neuronal cells are relatively resistant to m*Htt*-induced cell death [2]. To examine molecular-level differences that may underlie the pathophysiological observations, we studied differential transcriptomic response to *Htt* CAG expansion among the 5 surveyed tissues. To this end, we carried out statistical testing using a model that includes interaction of tissue with CAG length. We focused particularly on differences between striatum (strongly affected by HD), cortex (affected by HD to a lesser extent than striatum), and cerebellum (largely unaffected by HD). We identified 109 microRNAs with significant evidence of different DE between striatum and cerebellum and 57 microRNAs with significant evidence of different DE between striatum and cortex (S3 and S4 Tables).

Because the strength of transcriptional response (e.g., as measured by fold changes) is, in general, strongest in striatum, many of the microRNAs that are strongly associated with CAG length in the striatum also show significant evidence for inter-tissue differences in association with Q between the striatum and other tissues. To avoid essentially duplicating the list of microRNAs with strong DE in the striatum, we further prioritized those microRNAs whose fold change with respect to Q has the opposite sign in striatum and cortex or cerebellum, and whose associations with Q in both compared tissues (striatum and cortex or striatum and cerebellum) pass a suitable significance threshold (p<0.05 or FDR<0.05).

In comparing striatum vs. cerebellum, we found 15 microRNAs with significant difference in association with Q, opposite sign of association with Q in striatum and cerebellum, and FDR<0.05 for association with Q in both tissues. These 15 microRNAs include *Mir128* (in both -1 and -2 forms) that has been implicated in regulating motor behavior by modulating neuronal signaling networks and excitability in adult neurons [30]; *Mir218*, *Mir369* and *Mir543*. The last two are part of a cluster implicated in the fine-tuning of N-cadherin expression level and in the regulation of neurogenesis and neuronal migration in the developing neocortex [31].

In comparing striatum vs. cortex, we found a single microRNA, *Mirlet7f-1*, with significant difference in association with Q, opposite sign of association with Q in striatum and cortex, and FDR<0.05 for association with Q in both tissues, and additional 6 microRNAs (*Mir206*, *Mir301b*, *Mir92b*, *Mir378b*, *Mir208b*, *Mir449a*) satisfying p<0.05 for association with Q in both tissues. *Mir92b* has been implicated in the development of intermediate cortical progenitors in embryonic mouse brain [32], pointing again to a possible connection between CAG-length dependent dysregulation and developmental processes.

### MicroRNA correlations with, and target enrichments in, gene co-expression modules highlight developmental gene modules and their putative microRNA regulators

We next evaluated possible connections between microRNA and mRNA expression levels. A comprehensive analysis of mRNA profiling from the same samples has been reported previously [14]. The mRNA analysis utilized Weighted Gene Co-expression Network Analysis (WGCNA) [33,34] to define gene co-expression modules, i.e, clusters of genes whose expression profiles are correlated. Expression profiles of genes in each cluster were summarized using a representative profile called the eigengene [35]. By relating the module eigengene to CAG length the authors of [14] found 13 striatum and 5 cortex modules whose expression is strongly CAG length-dependent.

We first evaluated the enrichment of the CAG length-dependent mRNAmodules in predicted targets of significantly differentially expressed microRNAs. Specifically, we used predictions generated by MicroRNA.org [36], microCosm [37], targetScan [38], as well as a set of experimentally verified microRNA-mRNA interactions compiled in mirTarBase [39].

We found 124 striatum and 10 cortex microRNA-module combinations showing enrichment p-values less than 0.05; 78 of the 124 striatum combinations also passed the Bonferroni-corrected p-value threshold of 0.05 (S5 Table). In general, increased microRNA expression is expected to result in decreased mRNA expression of its target [40] although not all microRNAs impact the levels of their target mRNA. Hence, we further restricted our attention to those microRNA-module combinations where the microRNA expression levels are negatively correlated with the mRNA module eigengene. Fifty-five (44%) of the 124 striatum and 9 of the 10 cortex microRNA-module combinations exhibited negative correlations (mean of 6- and 10-month correlations in Series 1 data).

**Fig 5:**
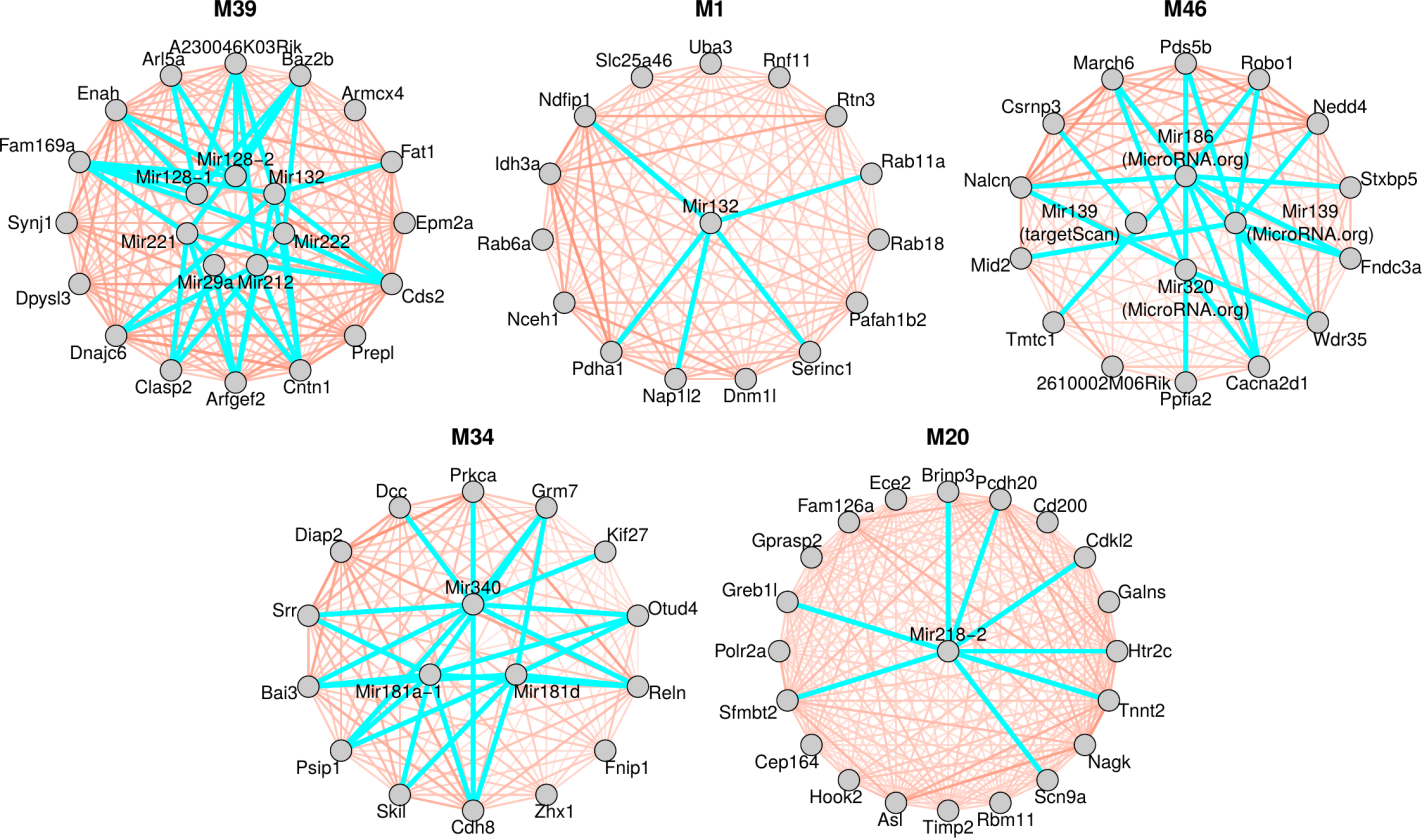
Network plots of top hub genes in CAG length-dependent modules and their putative regulator microRNAs. Each panel shows the top hub genes in one of the CAG length-dependent modules and microRNAs that are negatively correlated with the module eigengene and whose predicted targets are significantly enriched in the module. Predicted microRNA-target relationships are indicated by turquoise lines while the gene-gene co-expression relationships are indicated by red lines (thicker and wider lines indicate higher Topological Overlap). Only modules and microRNAs from Table 1 are shown whose correlations are less than -0.4; correlations of all mRNA-microRNA pairs shown in this figure are negative.

The microRNA-module correlation and enrichment analysis provides several intriguing findings (Table 1 and Fig 5). The strongest negative microRNA-module correlations were observed between striatum module M39 and *Mir132* (mean correlation -0.79) and *Mir212* (mean correlation -0.75); module M39 is also strongly enriched in predicted targets of both microRNAs (p = 1×10^−7^ for targets predicted by MicroRNA.org). Other examples of strongly negatively correlated microRNA-module pairs with strong enrichment of predicted targets include *Mir132* and module M1 (correlation -0.63, enrichment p-value 2×10^−6^), *Mir181d* and module M34 (correlation -0.58, enrichment p-value 4×10^−6^), and *Mir128* and module M39 (correlation -0.58, enrichment p-value 1×10^−7^). Modules M34 and M39, as well as modules M20 and M46 that also appear in Table 1, are enriched several development-related biological functions and in cadherin and protocadherin genes. These results provide hints that the upregulation of the developmental genes observed in these knock-in mice by [14] is also reflected by the microRNA transcriptome.

**Table 1:** MicroRNA-module pairs with strongest negative correlations and significant (FDR<0.05) enrichment of the module in predicted microRNA targets.

miRNA	mRNA module	miRNA-ME correlation^1^	Target source	Enrichment p value	Enrichment FDR
*Mir132*	39	-0.79	MicroRNA.org	9.6E-08	2.8E-06
*Mir212*	39	-0.75	MicroRNA.org	9.8E-08	2.9E-06
*Mir132*	1	-0.63	MicroRNA.org	2.2E-06	4.0E-05
*Mir181d*	34	-0.58	MicroRNA.org	4.1E-06	6.8E-05
*Mir128-2*	39	-0.58	MicroRNA.org	1.3E-07	3.6E-06
*Mir128-1*	39	-0.54	MicroRNA.org	1.3E-07	3.6E-06
*Mir221*	39	-0.51	MicroRNA.org	6.7E-07	1.4E-05
*Mir218-2*	20	-0.49	MicroRNA.org	1.7E-04	1.7E-03
*Mir29a*	39	-0.47	MicroRNA.org	3.2E-07	7.6E-06
*Mir181a-1*	34	-0.46	MicroRNA.org	1.9E-06	3.5E-05
*Mir186*	46	-0.46	MicroRNA.org	7.2E-10	4.5E-08
*Mir320*	46	-0.43	MicroRNA.org	3.4E-07	8.1E-06
*Mir222*	39	-0.43	MicroRNA.org	5.7E-08	1.8E-06
*Mir139*	46	-0.43	MicroRNA.org	3.0E-09	1.5E-07
*Mir139*	46	-0.43	targetScan	8.7E-04	4.8E-02
*Mir340*	34	-0.42	MicroRNA.org	8.7E-07	1.8E-05
*Mir203*	25	-0.39	MicroRNA.org	1.2E-03	8.7E-03
*Mir543*	1	-0.38	MicroRNA.org	5.1E-06	8.3E-05
*Mir186*	39	-0.38	MicroRNA.org	1.0E-11	1.3E-09
*Mir363*	39	-0.37	MicroRNA.org	1.4E-06	2.7E-05

^1^Correlation of the microRNA with the module eigengene (ME) which is a representative expression profile of the mRNA module.

### Relationship to prior microRNA studies

MicroRNA expression in HD has received relatively little attention from high-throughput studies to date. A previous study has examined microRNA expression in Brodmann Area 9 of the cortex in post-mortem brains from HD patients, pre-symptomatic carriers and controls [12]. Although this study is not directly comparable to ours, we have examined the concordance of association with CAG length in the cortex in our study and differential expression between HD patients and controls reported by [12].

Overall, we find a weak but significant correlation of significance Z statistics between the 10-month cortex and the human BA9 data (r = 0.26, p = 0.02 in Series 1 and r = 0.34, p = 0.003 in Series 2; S4 Fig). Correlations of 6-month cortex Z statistics were not significant. We found 8 microRNAs (*Mir615*, *Mir135b*, *Mir212*, *Mir132*, *Mir20a*, *Mir708*, *Mir99a*, *Mir138-2*) that are significantly (FDR<0.05) associated with CAG length in the meta-analysis of Series 1 6 and 10-month data and pass the p-value threshold of 0.05 in the human BA9 data. A single microRNA, *Mir615*, passes FDR<0.05 threshold in both human and mouse data. Hoss et al highlighted *Mir10b* in particular; this microRNA has increased expression with CAG length in 10-month data, concordant with the human results, but moves in the opposite direction in both 6-month data sets (S4 Fig).

Several other, smaller studies [9–11] have been published but their low sample sizes make direct comparisons with our data difficult. Such convergence of microRNA dysregulation in both HD mouse and patient brains could help to prioritize the study of this short list of microRNAs in HD pathogenesis.

## Discussion

While the transcriptome of messenger RNA has been extensively studied in both HD patients and in model organisms, comparatively little is known about the microRNA transcriptome in HD. This study is the first to extensively elucidate *Htt* CAG length-dependent changes in microRNA expression in the brain regions differentially vulnerable to HD. Our analyses of 4 brain regions–striatum, cortex, hippocampus and cerebellum–revealed CAG length-dependent microRNA expression changes, with a large number (159) of microRNAs progressively altered in striatum and smaller numbers in cerebellum (102), hippocampus (51) and cortex (45). In contrast, this progressive CAG length-dependent microRNA dysregulation was not observed in liver. Interestingly, the number of dysregulated microRNAs in cerebellum is about twice that in cortex and hippocampus, despite that fact that cerebellum is relatively unaffected by HD. This fact may reflect a robust transcriptional response that protects cerebellum from the deleterious effects of the CAG length expansion.

Clustering of tissues and time points by similarity of DE (Fig 4) also suggests that the transcriptional response in cerebellum is distinct from the other 3 brain regions. Because large numbers of significant associations in cerebellum were observed only in the highest Q length (Q175), we also cannot fully exclude the possibility that the cerebellum dysregulation is unique to long Q lengths, rather than being progressive with CAG length. However, fold changes in lower CAG lengths, especially in Q140 but to a lesser extent also in Q111 and Q92, are moderately correlated with fold changes in higher CAG lengths across time points and series (S5 Fig), supporting the progressivity of at least some of the transcriptional effects of CAG length expansion. We identified microRNAs whose transcriptional response to the CAG length expansion varies significantly among the 4 brain regions because these microRNAs may be involved in the differential sensitivity to CAG length expansion. We have specifically focused on those microRNAs whose differential expression in cerebellum, cortex or hippocampus is also significant and in the opposite direction relative to striatum. This analysis identified far more microRNAs in cerebellum (31) than in cortex (7) or hippocampus (3). This is another line of evidence that cerebellum exhibits a very specific transcriptional response to CAG length expansion.

Although a systematic enrichment analysis of the groups of microRNAs identified in the various analyses in GO terms has not revealed significant findings, individual microRNAs identified across our multiple analyses (differential expression, microRNA-module enrichment, differences in association with CAG length) provide several intriguing hints about mechanisms possibly involved in HD pathogenesis. The *Mir132*/*Mir212* cluster has been implicated in neuronal survival and shows strongest down-regulation in the striatum, followed by cerebellum and cortex. Several microRNAs (*Mir128*, *Mir181d*, *Mir92b*) have been implicated in regulation of neuronal development and differentiation; several of the top mRNA modules enriched in targets of the microRNAs are also enriched in developmental genes. A unique feature of the microRNA expression data presented here is that mRNA expression has also been profiled in the same animals [14]. This allowed us to identify microRNA-gene module pairs that are negatively correlated and the gene module is enriched in predicted targets of the microRNA, providing another layer of information about possible targets of dysregulated microRNAs (Table 1, Fig 5). Interestingly, top mRNA modules (M34, M39, M46, and M20) with strongest negative microRNA-module correlations and enrichment in microRNA targets are also enriched in developmental genes (e.g., cadherins and protocadherins). Our results suggest that CAG length-dependent dysregulation of microRNAs regulating gene expression during development could be causing (at least partially) the dysregulation of developmental gene expression. Future mechanistic studies will be necessary to test this hypothesis further.

Our comparison of microRNA changes in HD mouse model and patients helped to identify four microRNAs that are dysregulated in a similar manner. Together, such integrative systems level analyses will facilitate the prioritization and functional validation of microRNAs for their causal roles in HD pathogenesis.

We carried out the sequencing in two separate series of knock-in mice. Although the two designs are not identical and the lack of Q175 samples in the second series makes the significance of association with CAG length weaker, the second series nevertheless provides a useful tool for assessing validation, as well as allowing us to study a relatively short CAG length (Q50). Differential expression in higher Q samples (Q140) is robustly preserved in striatum and to a lesser degree in cerebellum and cortex. Differential expression in lower CAG lengths (specifically, Q92) is moderately preserved in the striatum, while the lack of concordance in cortex, cerebellum and liver indicates that lower CAG lengths have a much weaker effect in these tissues.

Our study also has several important limitations. Our validation data, although from different animals, come from the same mouse lines as the discovery data, and both data sets were generated using the same technology. Our study focused on surveying the small RNA transcriptome only; although we found intriguing hints of possible functional impact of the observed microRNA expression dysregulation, future detailed studies will be necessary to more clearly delineate and validate such functional effects.

In summary, our study provides an unbiased transcriptional characterization of the impact of CAG repeat expansion in endogenous murine *Htt* in vivo, and identifies microRNAs that are dysregulated in a CAG length- and age-dependent manner in specific brain regions. Our findings are available online in the HD resource HDinHD and should facilitate the future mechanistic understanding the role of microRNA in HD pathogenesis and therapeutics.

## Materials and methods

### Animal breeding and husbandry

The genotype lines as well as breeding of animals has been described in [13,14]. Briefly, this study utilized 7 heterozygous (HET) *Htt* KI lines expressing CAG repeats of 20 (*Htt*^tm2Mem^), 50 (*Htt*^tm3Mem^), 80 (*Htt*^tm1.1Pfs^), 92 (*Htt*^tm4Mem^), 111 (*Htt*^tm5Mem^) [41], 140 (*Htt*^tm1Mfc^) [42], and 175 (obtained by spontaneous CAG expansion in the Q140 model) [43]. For each one of the 7 lines, male HET mice were crossed with C57BL/6J female mice. Experimental animals were selected according to the following guidelines: No more than 1 animal per sex per genotype was selected from each litter. Animals originated from litters that could contribute to the experimental group with 4 animals were preferred over litters contributing 3, which in turn were preferred over litter contributing 2 animals. Body weight cut off: experimental animals had to weigh more than 11 g (females) and more than 13 g (males) by 5 weeks of age (the re-housing week).

Final experimental cages housed 8–10 animals in rat Opticages (Animal Care Systems, Inc.), about half HET and half WT, same sex, and originating from 10 different litters. Mice were fed 5001 rodent diet (Harlan-Teklad). On the week of arrival, one tail snip was collected for genotype confirmation, and electronic transponders (Data Mars) were implanted. One week after arrival, mice were handled twice for about 1 minute each. The first cage change was scheduled around 10 days following arrival to minimize disturbance of the cages that could trigger fighting.

The cages were maintained on a 12:12 light/dark cycle, with white light during the day and red light during the night, maintaining a low subjective light level for the subjects during the night period. While inside the cage, water was only available from within the IntelliCage corners, while food was freely available on the cage floor at all times.

### Ethics statement

This study was carried out in strict accordance with the recommendations in the Guide for the Care and Use of Laboratory Animals, NRC (2010). The protocols were approved by the Institutional Animal Care and Use Committee of PsychoGenics, Inc., an AAALAC International accredited institution (Unit #001213).

### Tissue selection

Striatum, cortex, hippocampus, cerebellum, and liver were selected for full profiling in Series 1. Specifically, at each of 3 time points (2, 6, 10 months), four female and four male heterozygous mice from each of 6 CAG repeat lengths (Q20, Q80, Q92, Q111, Q140 and Q175) were profiled, resulting in 48 samples from each tissue and each time point. Additional samples from wild type littermates from the Q20 line were profiled as well (striatum, hippocampus and cerebellum at all 3 ages, cortex and liver at 2 and 10m only). For Series 2, 6- and 10-month striatum, cortex, cerebellum and liver were profiled. Profiled CAG lengths included 20, 50, 92 and 140, in addition to wild type littermates of the Q20 and Q50 mice. As in Series 1, 4 female and 4 male animals were profiled from each group.

Tissue specimens were removed, flash frozen on dry ice, and RNA was harvested using the Qiagen miRNeasy kit. RNA quality and integrity were monitored via Agilent Bioanalyzer. A minimum of 3.75 μg of total RNA were used to collect enriched RNA subfractions for library construction. Libraries were sequenced on an Illumina HiSeq2000 sequencer using strand-specific, single end, 36-mer sequencing protocols to a minimum read depth of 5 million reads per sample. Clipped reads were aligned to mouse genome mm10 using the STAR aligner [44] using default settings. Read counts for individual genes were obtained using HTSeq [45].

### Data availability

All of our transcription data are available at Gene Expression Omnibus (Series 1 striatum: GSE65773; cortex: GSE65769, hippocampus: GSE73507, cerebellum: GSE73505, liver: GSE65771; Series 2 striatum: GSE78793, cortex: GSE78791, liver: GSE78792, cerebellum: GSE78790) and our online tool, HDinHD (www.HDinHD.org).

### Removal of potential outliers in expression data

Although differential analysis testing was carried out using the raw counts, for outlier removal we transformed the raw counts using variance stabilization. Within each tissue and time point we constructed Euclidean distance sample networks [46] and removed samples whose standardized inter-sample connectivity Z.k was below the threshold of -5. In this step we used robust standardization, i.e., subtracting the median and dividing by the (asymptotically consistent) median absolute deviation.

Initial differential expression testing indicated that despite removal of suspected outlier samples, measurements of individual microRNAs still contained apparent outliers (different samples appeared to be outliers for different microRNAs). Because removing all such outliers would lead to sample sizes that are too small, and since at the time of this analysis DESeq2 did not provide facilities to weigh individual samples, we have replaced suspected individual outliers as follows. First, for each microRNA (observation) and each tissue/time combination, we calculate Tukey bi-square-like weight coefficients λ based on the variance-stabilized transformation of the raw counts. Specifically, for each observation *x*, the coefficients are calculated as

$$\lambda ={\left(1-u^{2}\right)}^{2},$$

where *u* = min(1,|*x*−*m*|/(9*MAD*)), and *m* and *MAD* are median and median absolute deviation of the observations (one microRNA in one tissue and at one time point). These coefficients are used as weights when calculating the robust bi-weight midcorrelation [47]; the coefficients are near 1 for observations near the median, decrease as observations deviate more from the median, and are zero when the deviation is higher than 9 median absolute deviations (medians of the absolute deviations |*x*−*m*|). For each microRNA and tissue/time combinations, *MAD* is adjusted such that (1) 10^th^ percentile of the coefficients λ is at least 0.1 (that is, the proportion of observations with coefficients <0.1 is less than 10%) and (2) for each individual CAG length, 40^th^ percentile of the coefficients λ is at least 0.9 (that is, at least 40% of the observation have a high coefficient of at least 0.9). Finally, observations *x* with coefficients *λ*<0.5 are replaced by a weighted average of the original observation and the average $\bar{x}$ of all non-outlier observations (i.e., observations with *λ*≥0.5),

$$x^{replaced}=\left[\frac{(0.5-\lambda )\bar{x}+\lambda x}{0.5}\right],$$

where the square brackets indicate rounding to the nearest integer. Thus, mild outliers (0<*λ*<0.5) are replaced by a weighted average that weighs the average $\bar{x}$ of all non-outlier observations more as the outlier becomes more extreme, and the extreme outliers (*λ* = 0) are replaced completely by the average $\bar{x}$. This approach is similar in spirit to the outlier replacement implemented in DESeq2; our method has the advantage of explicitly preserving at least a few observations at each CAG length as non-outliers.

### Differential expression and association testing

Starting from the outlier-replace counts, we have used standard differential analysis and association testing via negative-binomial general linear models implemented in R package DESeq2 [48], with CAG length (Q) as a variable of interest and gender and batch as covariates. For testing with binary outcomes (e.g., Q140 vs. controls) we used Q20 and WT samples as controls. For analysis of association with CAG length as a numeric variable, we have used CAG length Q = 7 for the WT controls.

Differential expression analysis for binary genotype comparisons and association analysis for Q treated as a numeric variable are performed in DESeq2 using the same type of moderated negative binomial generalized linear models (GLMs). For DE analysis, the genotype is a binary variable while for association analysis, CAG length is treated as a numeric (continuous) variable. In DE analysis the resulting GLM coefficient is interpreted as log_2_ fold change, while for association analysis the coefficient represents thelog_2_ fold change per unit CAG length. MicroRNAs for which the coefficient is significantly (after Benjamini-Hochberg False Discovery Rate, FDR, correction) non-zero are considered significantly differentially expressed or associated with the relevant genotype comparison.

We have used the Wald test and disabled independent filtering and outlier replacement in DESeq2. We have run differential expression tests in each tissue and time point separately and combined results across the 6- and 10-month time points using meta-analysis. Resulting p-values were corrected for multiple comparisons using the Benjamini-Hochberg FDR method [49].

Since we have observed very few significant associations at 2 months, for testing of tissue-CAG length interactions we have combined the 6- and 10-month data and added age as a covariate. To determine significance, we have used the likelihood ratio test (LRT) where the full and reduced models differ by the addition of a CAG length–tissue interaction term.

### Meta-analysis

Since the DE and association statistics show strong concordance between 6 and 10 month data, we pool association statistics across the 2 time points (data sets) in each tissue using meta-analysis. A simple, yet powerful meta-analysis method relies on combining the Z statistics from individual data sets [50,51]. Specifically, for each microRNA *i* and data set *a*, one obtains a Z statistic *Z*_*ia*_, for example, by the inverse normal transformation of the p-value. Next, a meta-analysis *Z*_i_ statistic for microRNA *i* is calculated as

$$Z_{i}=\frac{1}{\sqrt{N_{sets}}}\overset{N_{sets}}{\underset{a=1}{\sum }}Z_{ia}$$

Here *N*_*sets*_ *=* 2 and the index *a* runs over the 6 and 10-month data sets for a given tissue. The meta-analysis statistic *Z*_*i*_ is approximately normally distributed with mean 0 and variance 1; the corresponding p-value is then calculated using the normal distribution.

### Permutation tests of significance

When correlating two vectors of microRNA significance statistics (e.g., for studying concordance of differential expression), the standard correlation p-value is biased in the anti-conservative direction (inflated significance). This occurs because the individual microRNAs are not all independent, violating the assumption of independence that underlies the calculation of standard correlation p-value. To arrive at realistic p-values for concordance of DE, we repeated each DE calculation 200 times with randomly permuted genotype values. For each observed DE correlation *r*, we then calculated the corresponding 200 correlations *r*_*i*_, *i* = 1,2,…,200 of significance statistics from permuted DE test as well as their mean ${\bar{r}}_{perm}$ and standard deviation *s*_*perm*_. An approximate, semi-parametric p-value can then be obtained by calculating the permutation Z statistic

$$Z_{perm}=\frac{r-{\bar{r}}_{perm}}{s_{perm}},$$

and calculating the corresponding two-sided p-value using the normal distribution function. We emphasize that the p-values obtained in this manner are approximate but are expected to be much closer to unbiased than the naïve Student correlation p-values.

### MicroRNA clusters

MicroRNA clusters are small groups of microRNAs whose genomic locations are very close and that often share a common regulatory mechanism and expression pattern [52]. To facilitate the interpretation of our results in terms of clusters, we have defined microRNA clusters as groups of microRNAs whose genomic positions are at most 200kb apart. We report the microRNA cluster membership information in our comprehensive result tables (S1, S2 and S4 Tables), and Figs 1 and 2 contain not only the numbers of significant microRNAs but also the number of distinct clusters represented by the microRNAs.

## Supporting information

S1 Fig Overlaps of DE microRNAs among all comparisons in striatum. For each pair of differential expression analyses, the table shows the overlap of the significantly (FDR<0.05) differentially expressed microRNAs in the two analyses and the corresponding hypergeometric p-value. The color scale indicates the number of overlapping DE microRNAs as a fraction of the minimum of the numbers of DE microRNAs in the two analyses. The diagonal shows the number of DE microRNAs in each comparison.
(PDF)

S2 Fig Concordance of Series 1 and Series 2. For each of the 4 tissues and 3 analyses present in both series, a scatterplot shows the meta-analysis DE significance in Series 2 (y-axis) vs. 6- and 10-month meta-analysis in Series 1. Each dot represents a single microRNA. Correlations and the corresponding permutation-based p-values are shown in the title of each plot. The correlations serve as a measure of concordance of DE between Series 1 and Series 2.
(PDF)

S3 Fig Statistical significance of concordance of association with Q as a numeric variable across tissues and time points. This figure shows the permutation-based p-values corresponding to the correlations shown in Fig 3.
(PDF)

S4 Fig Concordance of DE in our cortex data with the results of Hoss et al. In each panel, the x-axis shows the microRNA significance Z statistic for continuous Q in one of our 6 or 10 month cortex data sets, and the y-axis shows the significance Z statistic for association with disease status in human BA9 data [12]. Each point represents a single microRNA. Correlations and the corresponding permutation-based p-values are shown in the title of each panel.
(PDF)

S5 Fig Concordance of DE across all tests in cerebellum. For each of the DE analyses carried out on cerebellum data, the table shows the correlations of DE significance Z statistics and the corresponding semi-parametric permutation-based p-values. Only correlations whose permutation p-value is less than 0.05 are shown explicitly. Color scale indicates the correlation value.
(PDF)

S1 Table Complete results of association screening of individual microRNAs. Each sheet in the file corresponds to one data set (tissue and series). Each sheet contains meta-analysis statistics, mean expression and differential expression statistics for binary comparisons of higher CAG length samples vs. controls (e.g., suffix Q80.vs.ctrl corresponds to comparison of Q80 vs. controls) and as well as association statistics for CAG length (Q) treated as a continuous variable.
(XLS)

S2 Table Counts of significantly associated and validated microRNAs. For each of the 4 tissues for which there are validation (Series 2) data, the table lists the number of microRNAs significantly (FDR<0.05) associated with CAG length in Series 1 data, and numbers of those of the significantly associated microRNAs that validate (i.e., pass the significance threshold) in Series 2. Two significance thresholds are used for validation, FDR<0.05 and p<0.05. The numbers and fractions are further split according to the direction (up or down with CAG length) in the discovery (Series 1) data.
(CSV)

S3 Table Numbers of microRNAs with significant (FDR<0.05) tissue-CAG length interaction (TQI). The third column indicates the number of the microRNAs for which there is no significant evidence of a change of direction (sign) of association with CAG length: the associations with CAG length either have the same sign or at least one did not pass the p<0.05 threshold. The 4^th^ and 5^th^ columns give the numbers of microRNAs with opposite signs of association with CAG length that also pass the indicated significance thresholds in both compared tissues; we consider this a significant evidence of opposite direction of transcriptional response to CAG length mutation.
(DOCX)

S4 Table Statistics testing for tissue-CAG length interactions (differences in CAG association between tissues). Each sheet in the file corresponds to one pairwise tissue interaction and contains association statistics for interaction as well as relevant statistics of association with Q as a continuous variable in each tissue. Column significanceIndex is 0, 1 or 2 if the microRNA is significantly associated with CAG length in neither, one or both tissues, respectively. Column exprDivergesInHigherQ is 1 if the expression difference between the two tissues increases with increasing CAG length.
(XLS)

S5 Table Enrichment mRNA modules in predicted targets of microRNAs. For each microRNA, this table summarizes mRNA modules that are enriched in the predicted targets of the microRNA. The mRNA modules were identified in mRNA data from the same mice; the analysis is described in [14]. Columns are annotated in a separate sheet in the file.
(XLS)
