# Supplementary figures and images for: MicroRNA signatures of endogenous Huntingtin CAG repeat expansion in mice
Source: PLoS One. 2018 Jan 11;13(1):e0190550. doi: 10.1371/journal.pone.0190550 (PMC5764268; doi:10.1371/journal.pone.0190550)

Overlaps of significant miRNAs, Striatum

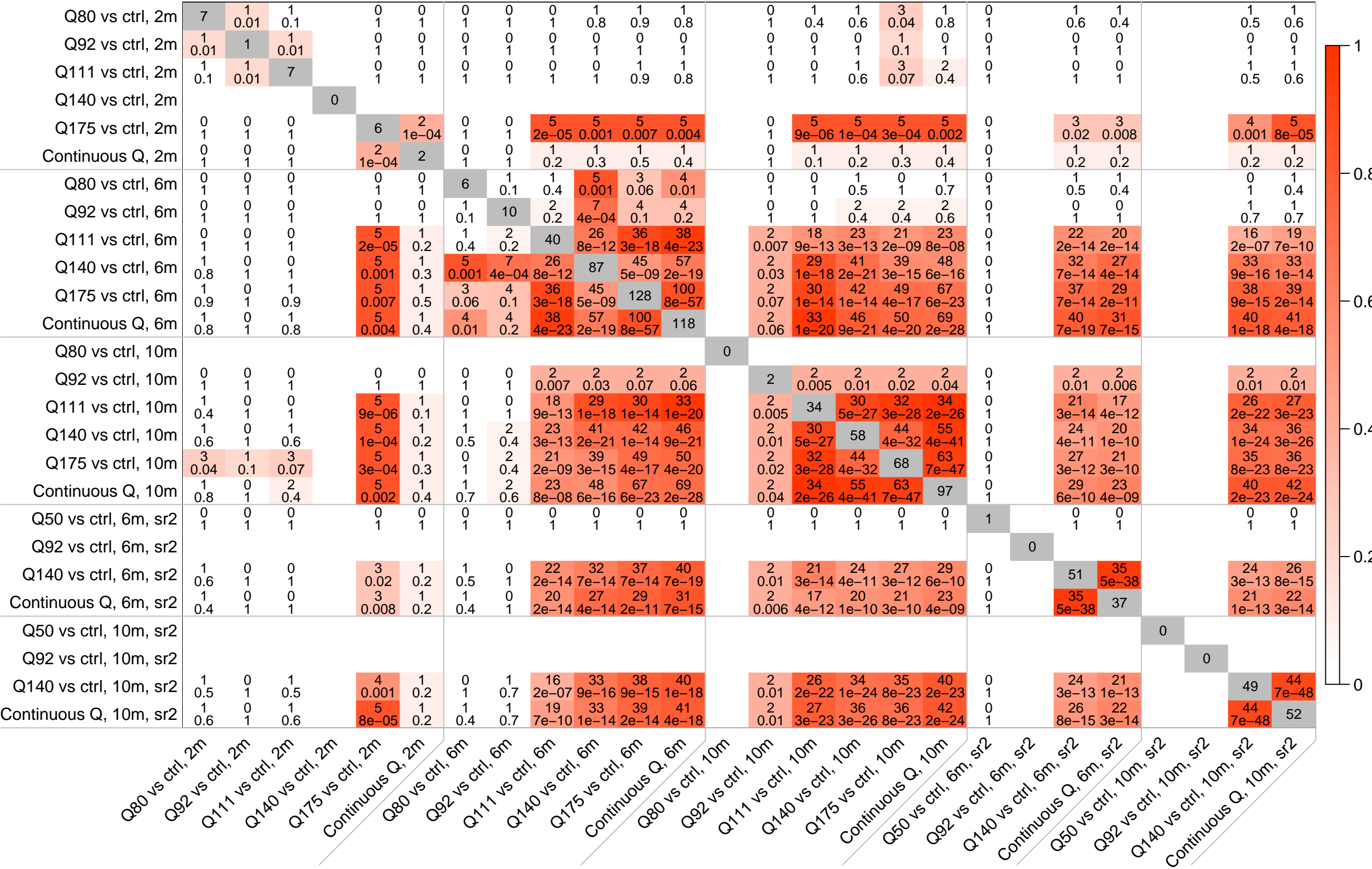

Supplement: S1 Fig — For each pair of differential expression analyses, the table shows the overlap of the significantly (FDR<0.05) differentially expressed microRNAs in the two analyses and the corresponding hypergeometric p-value. The color scale indicates the number of overlapping DE microRNAs as a fraction of the minimum of the numbers of DE microRNAs in the two analyses. The diagonal shows the number of DE microRNAs in each comparison. (PDF) [file pone.0190550.s001.pdf]

# Concordance of significance Z statistics for Q

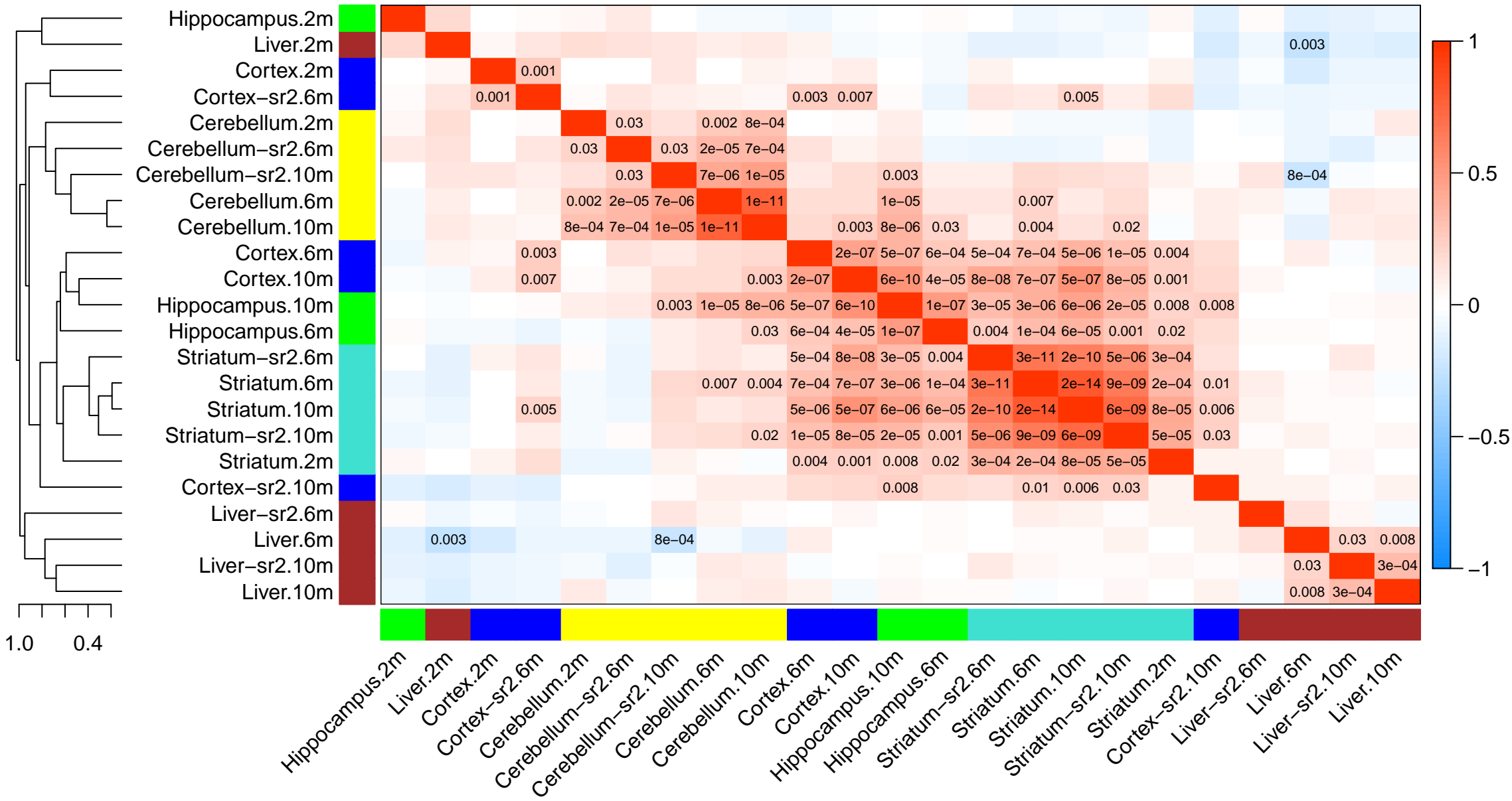

Supplement: S3 Fig — This figure shows the permutation-based p-values corresponding to the correlations shown in Fig 3. (PDF) [file pone.0190550.s003.pdf]

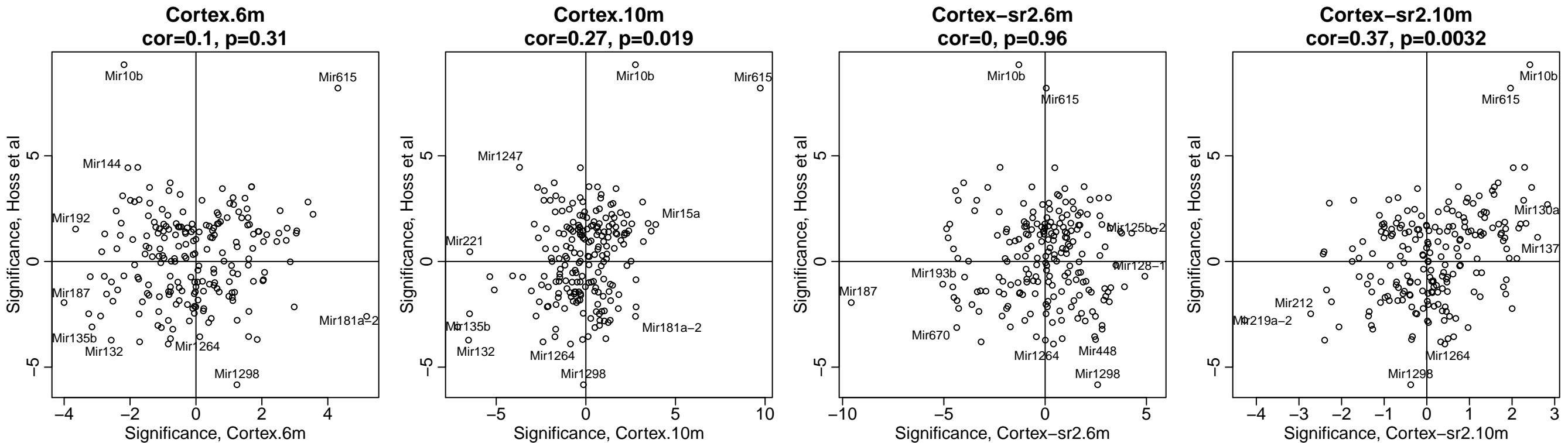

Supplement: S4 Fig — In each panel, the x-axis shows the microRNA significance Z statistic for continuous Q in one of our 6 or 10 month cortex data sets, and the y-axis shows the significance Z statistic for association with disease status in human BA9 data [12]. Each point represents a single microRNA. Correlations and the corresponding permutation-based p-values are shown in the title of each panel. (PDF) [file pone.0190550.s004.pdf]

Correlation of significance Z statistics, Cerebellum

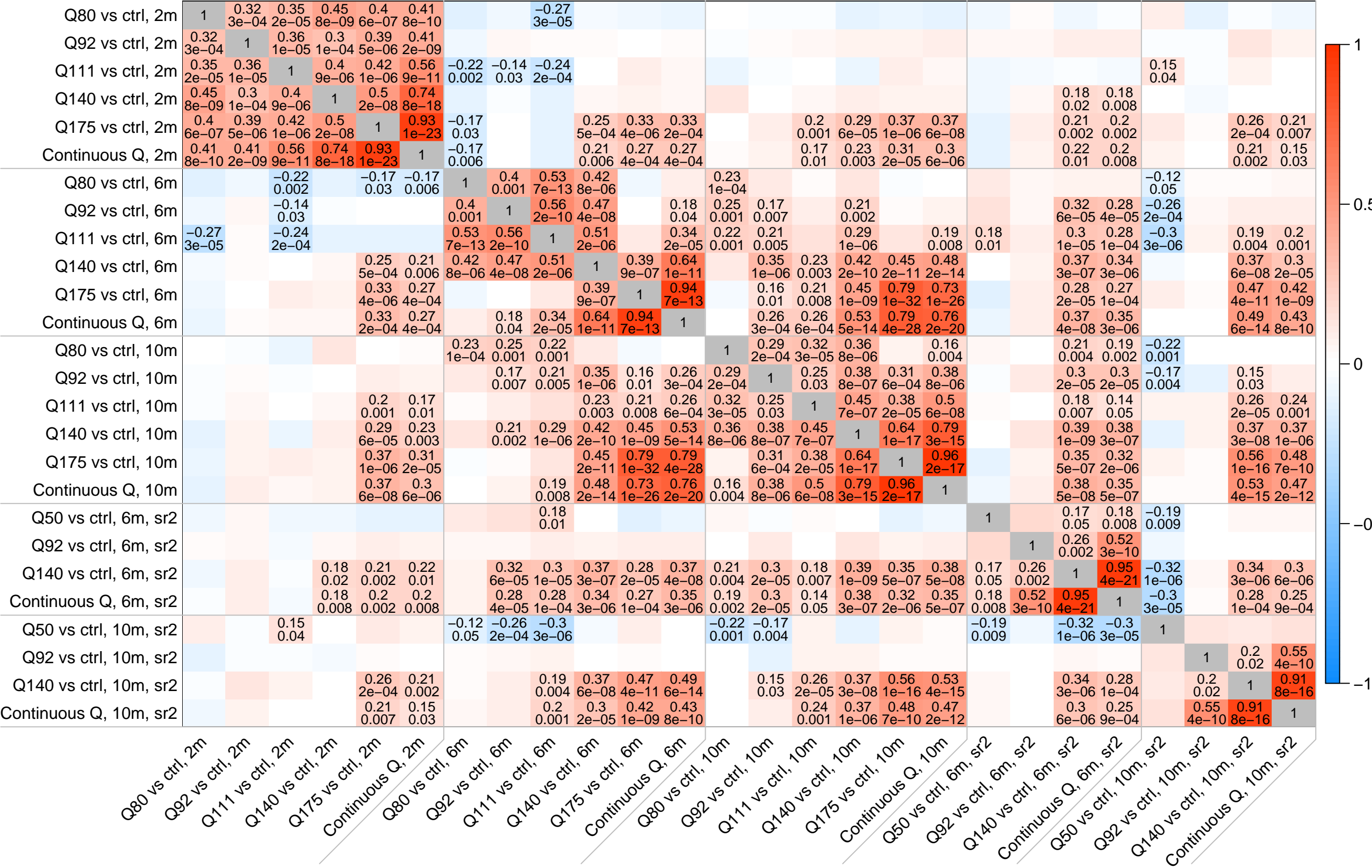

Supplement: S5 Fig — For each of the DE analyses carried out on cerebellum data, the table shows the correlations of DE significance Z statistics and the corresponding semi-parametric permutation-based p-values. Only correlations whose permutation p-value is less than 0.05 are shown explicitly. Color scale indicates the correlation value. (PDF) [file pone.0190550.s005.pdf]
