# Supplementary material for: MicroRNA signatures of endogenous Huntingtin CAG repeat expansion in mice
Source: PLoS One. 2018 Jan 11;13(1):e0190550. doi: 10.1371/journal.pone.0190550 (PMC5764268; doi:10.1371/journal.pone.0190550)
Supplement: S2 Fig — For each of the 4 tissues and 3 analyses present in both series, a scatterplot shows the meta-analysis DE significance in Series 2 (y-axis) vs. 6- and 10-month meta-analysis in Series 1. Each dot represents a single microRNA. Correlations and the corresponding permutation-based p-values are shown in the title of each plot. The correlations serve as a measure of concordance of DE between Series 1 and Series 2. (PDF) [file pone.0190550.s002.pdf]

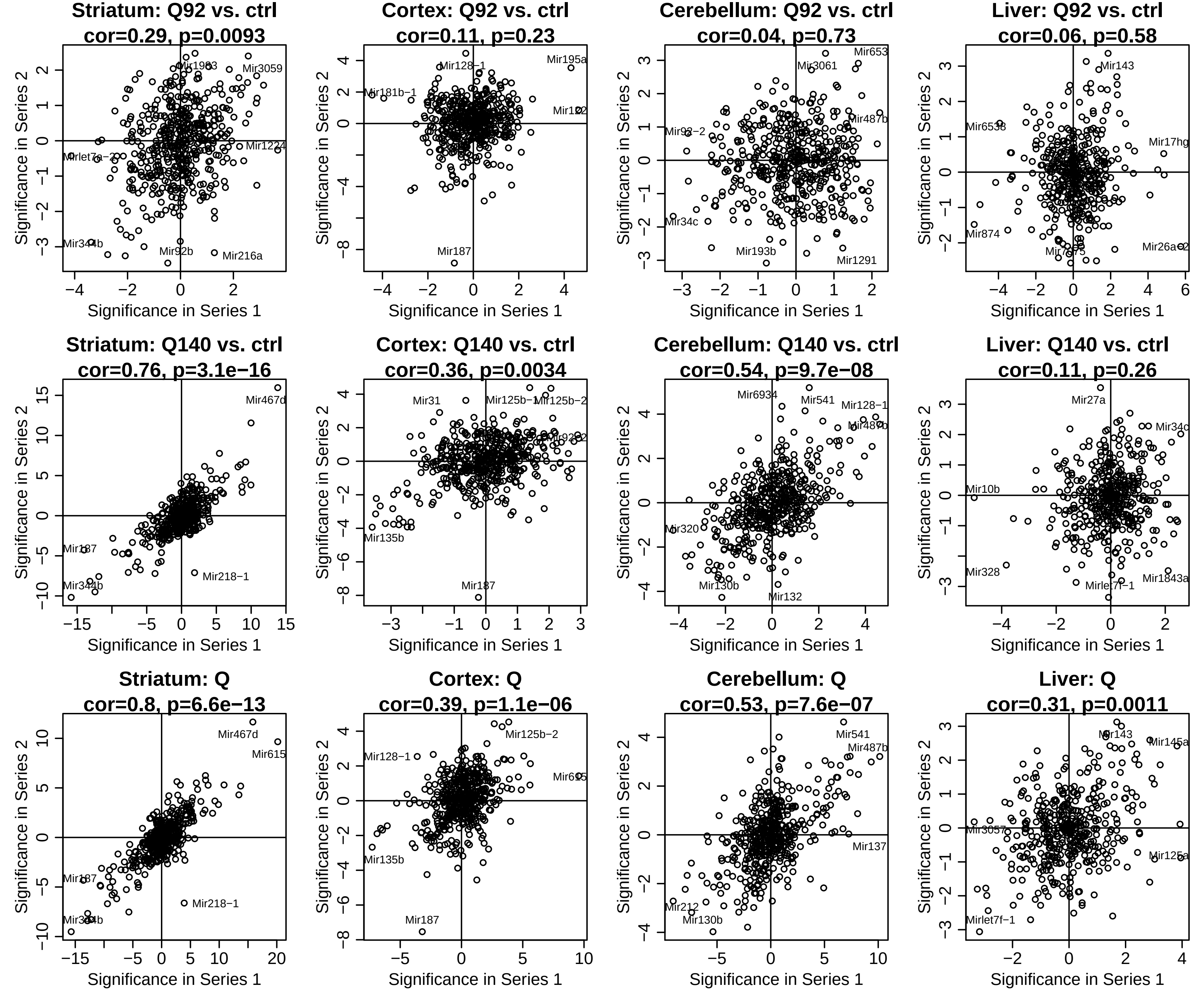

Striatum: Q92 vs. ctrl
cor=0.29, p=0.0093
Mir3059
Mir1224
Mirlet7a−2
Mir344b
Mir92b
Mir216a
Cortex: Q92 vs. ctrl
cor=0.11, p=0.23
Mir128−1
Mir195a
Mir181b−1
Mir187
Cerebellum: Q92 vs. ctrl
cor=0.04, p=0.73
Mir653
Mir3061
Mir487b
Mir92−2
Mir34c
Mir193b
Mir1291
Liver: Q92 vs. ctrl
cor=0.06, p=0.58
Mir143
Mir6538
Mir17hg
Mir874
Mir26a−2
Striatum: Q140 vs. ctrl
cor=0.76, p=3.1e−16
Mir467d
Mir187
Mir344b
Mir218−1
Cortex: Q140 vs. ctrl
cor=0.36, p=0.0034
Mir31
Mir125b−1
Mir125b−2
Mir135b
Mir187
Cerebellum: Q140 vs. ctrl
cor=0.54, p=9.7e−08
Mir6934
Mir541
Mir128−1
Mir487b
Mir320
Mir130b
Mir132
Liver: Q140 vs. ctrl
cor=0.11, p=0.26
Mir27a
Mir34c
Mir10b
Mir328
Mirlet7f−1
Mir1843a
Striatum: Q
cor=0.8, p=6.6e−13
Mir467d
Mir615
Mir187
Mir218−1
Cortex: Q
cor=0.39, p=1.1e−06
Mir125b−2
Mir128−1
Mir135b
Mir187
Cerebellum: Q
cor=0.53, p=7.6e−07
Mir541
Mir487b
Mir137
Mir212
Mir130b
Liver: Q
cor=0.31, p=0.0011
Mir143
Mir145a
Mir3057
Mir125a
Mirlet7f−1
Significance in Series 1
Significance in Series 2
