# Supplementary material for: MicroRNA signatures of endogenous Huntingtin CAG repeat expansion in mice
Source: PLoS One. 2018 Jan 11;13(1):e0190550. doi: 10.1371/journal.pone.0190550 (PMC5764268; doi:10.1371/journal.pone.0190550)
Supplement: S3 Table — The third column indicates the number of the microRNAs for which there is no significant evidence of a change of direction (sign) of association with CAG length: the associations with CAG length either have the same sign or at least one did not pass the p<0.05 threshold. The 4th and 5th columns give the numbers of microRNAs with opposite signs of association with CAG length that also pass the indicated significance thresholds in both compared tissues; we consider this a significant evidence of opposite direction of transcriptional response to CAG length mutation. (DOCX) [file pone.0190550.s008.docx]

**Supplemetary Table 4: Numbers of microRNAs with significant (FDR<0.05) tissue-CAG length interaction (TQI). The third column indicates the number of the microRNAs for which there is no significant evidence of a change of direction (sign) of association with CAG length: the associations with CAG length either have the same sign or at least one did not pass the p<0.05 threshold. The 4^th^ and 5^th^ columns give the numbers of microRNAs with opposite signs of association with CAG length that also pass the indicated significance thresholds in both compared tissues; we consider this a significant evidence of opposite direction of transcriptional response to CAG length mutation.**

| **Tissue comparison** | **Total miRNAs with significant TQI** | **Non-significant sign difference** | **Different sign, both p-values significant** | **Different sign, both FDRs significant** |
| --- | --- | --- | --- | --- |
| Striatum vs. Cortex | 57 | 50 | 6 | 1 |
| Striatum vs. Cerebellum | 109 | 78 | 16 | 15 |
| Striatum vs. Hippocampus | 63 | 60 | 1 | 2 |
| Striatum vs. Liver | 37 | 31 | 6 | 0 |
| Cortex vs. Cerebellum | 44 | 37 | 4 | 3 |
| Cortex vs. Hippocampus | 4 | 4 | 0 | 0 |
| Cortex vs.  Liver | 8 | 7 | 1 | 0 |
| Hippocampus vs. Cerebellum | 58 | 49 | 5 | 4 |
| Hippocampus vs. Liver | 5 | 4 | 1 | 0 |
| Cerebellum vs. Liver | 5 | 4 | 1 | 0 |
